# Generational Difference of Axial Length and Its Risk Factors in Urban and Rural China

**DOI:** 10.1155/2019/1607064

**Published:** 2019-11-26

**Authors:** Zhong Lin, Balamurali Vasudevan, Kenneth J Ciuffreda, Tie Ying Gao, Hong Jia Zhou, Yuan Bo Liang

**Affiliations:** ^1^The Affiliated Eye Hospital, School of Ophthalmology and Optometry, Wenzhou Medical University, Wenzhou, Zhejiang, China; ^2^College of Optometry, Mid Western University, Glendale, AZ, USA; ^3^Department of Biological and Vision Sciences, SUNY College of Optometry, New York, NY, USA; ^4^Handan Eye Hospital, Handan, Hebei, China

## Abstract

**Purpose:**

To compare the axial length difference (ALD) and the estimated generational axial length shift (ALS) from parents to their children and its risk factors in urban and rural China.

**Methods:**

Participants were enrolled from two longitudinal cohort studies, the Beijing Myopia Progression Study (BMPS) and the Handan Offspring Myopia Study (HOMS). Ocular biometry was performed in both parents and their children. ALD was defined as the difference between the children's axial length and the corresponding parental axial length. Generational ALS was estimated according to a binominal prediction model at 18 years of age.

**Results:**

237 and 380 urban and rural Chinese children (6–17 years) and their parents from the BMPS and HOMS, respectively, were enrolled. Children's axial length was estimated to be closest to the parental axial length at 11 and 9 years of age in the urban and rural areas, respectively; the estimated generational ALS would be 1.53 and 0.57 mm, respectively. Multivariable regression analysis revealed that older children (urban *β* = 0.26, *p* < 0.001; rural *β* = 0.11, *p* < 0.001) and males had larger ALD (urban *β* = 0.55, *p* < 0.001; rural *β* = 0.52, *p* < 0.001) in both areas. Furthermore, urban children with more educated parents (fathers: *β* = −0.30, *p*=0.002; mothers: *β* = −0.29, *p*=0.004) and more outdoor activity (*β* = −0.23, *p*=0.006) had a less ALD.

**Conclusions:**

The urban generational axial length shift was estimated to be approximately 1 mm longer than that of the rural area. These results suggest different environmental effects on the ocular development in these two populations of Chinese children.

## 1. Introduction

Myopia in school children is a major public health problem in both rural and urban populations in East Asia [1, 2]. Both genetic and environmental factors may play important roles in the development of myopia [3, 4]. The former is related to preprogrammed growth of the eye [3, 4]. The latter modulates and influences the development of myopia [3–6]. For example, recent studies from the Beijing Myopia Progression Study (BMPS) and the Handan Offspring Myopia Study (HOMS) found that the children's myopia was approximately 2D and 1D higher than their parental myopia at the age of 18 years in urban and rural areas of northern China, respectively [5, 6]. Different environment factors were believed to reflect their different generational myopia shifts [5, 6].

It has been reported that axial length is longer, vitreous chamber is deeper, lens is thinner, anterior chamber is deeper, and the cornea is flatter in myopes [7, 8]. Since the incidence of juvenile myopia is high in East Asia [1, 9], investigation of variations in the ocular parameters between generations may enable better understanding of both the mechanisms and risk factors that lead to eye growth and myopia.

The purpose of the present study was to describe the quantitative, age-specific, axial length difference (ALD), and its generational shift from parents to their children, as well as the risk factors related to ALD, in urban (BMPS) and rural (HOMS) areas of northern China. Other ocular parameter differences (OPDs), including central anterior chamber depth difference (ACDD), length thickness difference (LTD), and vitreous chamber depth difference (VCDD), are also reported.

## 2. Methods

### 2.1. Subjects

The BMPS was a three-year, hospital-based, cohort study that primarily aimed to investigate the possible relationship between near work-induced transient myopia (NITM) and myopia progression in Chinese children [10]. The HOMS was the offspring study of the Handan Eye Study [11], a population-based study conducted in Handan, Hebei Province of North China [12]. It is noteworthy that these two studies shared many of the same procedures (e.g., visual acuity, ocular biometry, and cycloplegic autorefraction) and questionnaires with BMPS [10, 12]. The BMPS and HOMS followed the tenets of the Declaration of Helsinki and were approved by the ethics committee of Beijing Tongren Hospital and Handan Eye Hospital, respectively. All participants (children and their parents) signed a written informed consent. Details of the study design, sample size estimation, and baseline characteristics of BMPS and HOMS were reported elsewhere [10, 12].

Children and their parents who completed the ocular biometry examination were included in this study. The exclusion criteria were as follows: (1) children or parents with amblyopia or strabismus; (2) children or parents with a history of intraocular surgery or penetrating ocular trauma; and (3) children or parents with significant medical or ocular health problems. The participants (both children and their parents) received comprehensive vision examinations and the detailed related questionnaire.

### 2.2. Ocular Biometry

Subjects lay supinely on the testing bed. One drop of 0.5% proparacaine HCL (Alcaine®, Alcon, TX) was instilled in each eye. They were then instructed to focus and fixate upon a red target on the ceiling directly above his/her head using the contralateral eye during the measurement. Ocular parameters including anterior chamber depth (ACD), lens thickness (LT), vitreous chamber depth (VCD), and axial length (AL) of both eyes were measured by a biometer A-scan (Axis-II PR (Quantel Medical, Clermont-Ferrand, France)). Ten values of each parameter were obtained, and the average value was used for further analysis. One drop of tobramycin 0.3% (Tobrex®, Alcon) antibiotic ointment was instilled in each eye after the measurements were completed.

### 2.3. Questionnaire

The questionnaire used in the Sydney Myopia Study [13] was translated into Chinese with minor modifications [12, 14–16]. Information regarding the child's near work/outdoor activity, books read per week, and living environment was assessed via this detailed questionnaire. The average hours spent on near work activity were totaled for drawing, homework, reading, and handheld computer use. Time spent on outdoor activities was evaluated on the basis of responses to queries about playing outdoors, family picnics and barbeques, bicycle riding, hiking, and outdoor sports. Activity levels were graded into three population tertiles of the average daily hours spent on these different activities [15–17].

### 2.4. Definitions

Children's ocular parameters were defined and quantified as the average of the right eyes, while the parental ocular parameters were defined and quantified as the average of the fathers' right eyes and mothers' right eyes. The OPDs were defined as the difference between the ocular parameters of the children and their parents, i.e., the children's OPDs minus their parental average OPDs.

### 2.5. Data Analysis

The data analysis methods in the current study were similar to our previous reports on generational myopic shift [5, 6]. Since the number of subjects in some age subgroups (i.e., children aged 15, 16, or 17 years) was less than 20, adjacent age groups were combined. The OPDs of each family were calculated and then averaged in each age group. The mean OPDs and proportion of children with longer AL than their parents as a function of the children's combined ages were calculated. Binominal fitting functions with ALD/proportion of longer AL of children than their parents and the children's combined ages as the dependent and independent variables, respectively, were fitted to investigate the trend of ALD and longer AL proportions. Considering there may be a cluster effect, since some of the families had more than one child, generalized estimating equations were performed to establish these fitting functions. The binominal fitting functions had the largest *R*^2^ and smallest quasi-likelihood under the independence model criterion as compared to other comprehensive fitting functions, such as linear or logarithmic functions. Generational axial length shift was defined as the estimated ALD according to the binominal fitting function at the age of 18 years, since the age of myopia stabilization in the majority of children was reported to be less than 18 years [18].

Data having a normal distribution were presented as the mean ± standard deviation and tested with the Student's *t*-test. Pearson correlation between the OPDs and the children's age was performed. Generalized linear models were performed to calculate the age- and gender-adjusted ALD and to determine the association between ALD and the putative risk factors assessed from the questionnaire. Statistical analysis was performed with Statistical Analysis System for Windows version 9.1.3 (SAS Inc., Cary, NC). A *p* value less than 0.05 was considered to be statistically significant.

## 3. Results

A total of 234 urban families (234 pairs of parents and 237 children) with completed ocular parameter data were enrolled. No family was excluded in the urban area. A total of 246 rural families (246 pairs of parents and 400 children) had completed ocular parameter data. Twelve families (12 pairs of parents and 20 children) were excluded due to presence of amblyopia, strabismus, or significant ocular history of a family member. Thus, 234 rural families (234 pairs of parents and 380 children) were enrolled. There were 121 boys (51.1%) and 210 boys (55.3%) in the urban and rural areas, respectively. Characteristics of the parents are also presented in Table 1.

The ACDD, VCDD, and ALD were positively correlated with the children's age in both the urban (*r* = 0.41, *p* < 0.001; *r* = 0.61, *p* < 0.001; *r* = 0.62, *p* < 0.001) and rural (*r* = 0.19, *p* < 0.001; *r* = 0.30, *p* < 0.001; *r* = 0.32, *p* < 0.001) areas.  Table 2 presents the ocular parameter differences between the parents and their children by the children's age in the urban and rural areas. Children had deeper ACD as compared to their parents in both areas at each age group (all *p* < 0.001). The ACDD increased from 0.21 ± 0.28 mm and 0.51 ± 0.29 mm in the urban and rural children at 6.0–7.9 years of age, respectively, to 0.54 ± 0.31 mm and 0.74 ± 0.31 mm at 16.0–17.9 years of age, respectively (both *p*_trend_ < 0.001). The urban children exhibited a shorter AL than their parents at age 6.0–9.9 years (both *p* < 0.001), no significant difference at 10.0–11.9 years (*p*=0.52), and had longer AL at 12.0–17.9 years old (all *p* < 0.001). Correspondingly, the rural children demonstrated a shorter AL than their parents at age 6.0–7.9 years (*p*=0.009), no significant difference at 8.0–9.9 years (*p*=0.43), and had longer AL at 10.0–17.9 years (all *p* < 0.01). The ALD increased from −0.96 ± 1.02 mm and −0.32 ± 0.74 mm in the urban and rural children at 6.0–7.9 years, respectively, to 1.17 ± 0.97 mm and 0.57 ± 0.73 mm at 16.0–17.9 years, respectively (both *p* < 0.001). Similar results were found for VCD in both areas. The LTD was negatively correlated with the children's age in both the urban (*r* = −0.30, *p* < 0.001) and rural areas (*r* = −0.12, *p*=0.02). Children showed thinner LT than their parents at each age group (all *p* < 0.001). The LTD decreased from −0.56 ± 0.22 mm at 6.0–7.9 years to −0.76 ± 0.19 mm at 16.0–17.9 years in the urban children (*p*_trend_ < 0.001) and −0.70 ± 0.31 mm to −0.84 ± 0.24 mm, correspondingly in the rural children (*p*_trend_=0.024).

**Figure 1:**
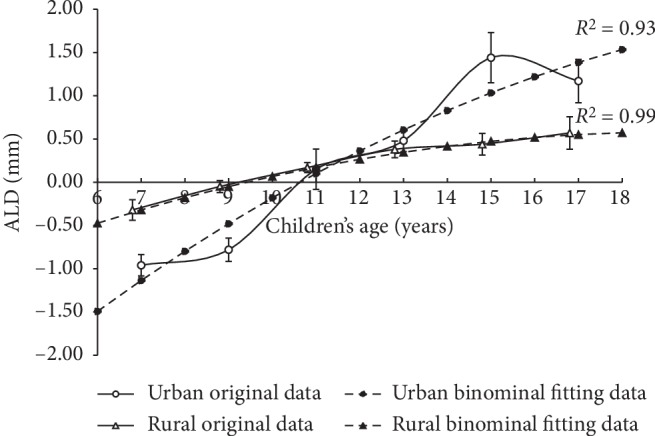
Original axial length difference (ALD) data from parents to children as a function of children's combined age in urban (*open circles*) and rural areas (*open triangles*), as well as their binominal fitting data (filled circles and filled triangles, respectively). The binominal fitting function in urban and rural areas was ALD = −0.0095*a*^2^ + 0.48*a* − 4.03 and ALD = −0.006*a*^2^ + 0.23*a* − 1.64 (*a* stands for the children's age), respectively. ALD was defined as children's axial length minus parental axial length; plotted is mean ± standard error. Children's combined age was defined as the average of every two adjacent ages. For the sake of clarity, the original rural ALD was shifted 0.2 units towards the left.

**Figure 2:**
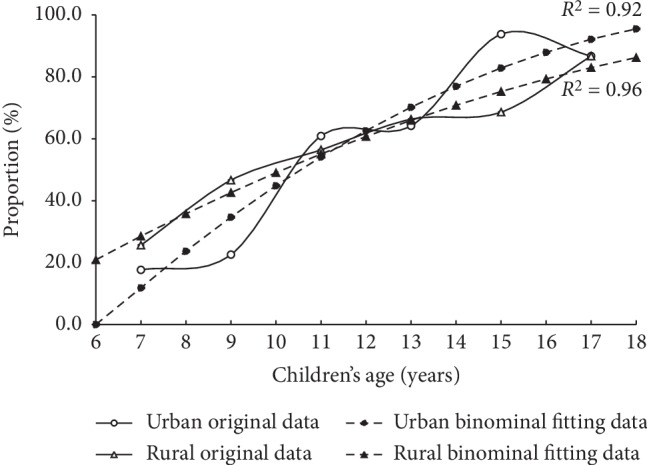
Proportion of children with longer axial length than their parents as a function of children's combined age in urban (*open circles*) and rural areas (*open triangles*), as well as their binominal fitting data (*filled circles and filled triangles, respectively*). The binominal fitting function in urban and rural areas was percentage = −0.42*a*^2^ + 18.19*a* − 94.72, and percentage = −0.20*a*^2^ +10.20*a* − 33.16, respectively (*a* stands for the children's age). Children's combined age was defined as the average of every two adjacent ages.

Using the binominal fitting functions, the children's AL would be closest to the average AL of their parents at the age of 11 (ALD = 0.10 mm) and 9 (ALD = −0.05 mm) years in the urban and rural areas, respectively. Furthermore, the ALD would be 1.53 mm and 0.57 mm at the age of 18 years in the urban and rural areas, respectively (Figure 1). The proportion of children having longer AL compared to the average of their parents also increased with the children's age in both areas (Figure 2). Approximately 17.7% (12/68) and 25.6% (10/39) of children at 6.0–7.9 years had a longer AL than their parents in the urban and rural areas, respectively. This increased to 86.7% (13/15) at 16.0–17.9 years in both areas. Furthermore, using a similar function, the estimated proportion would be 95.5% and 86.2% at the age of 18 years, respectively.

In the multivariable analysis, it was found that children who were older (urban *β* = 0.26, *p* < 0.001; rural *β* = 0.11, *p* < 0.001) and were male (urban *β* = 0.55, *p* < 0.001; rural *β* = 0.52, *p* < 0.001) had larger ALD in both the urban and rural areas, after adjusting for children's gender and age, respectively. After adjusting for both age and gender, children with more educated parents (fathers: *β* = −0.30, *p*=0.002; mothers: *β* = −0.29, *p*=0.004) and more time in outdoor activity (*β* = −0.23, *p*=0.006) had less ALD in the urban area. However, no significant association between ALD and near work time, books read, continuous reading time, and living environments in both urban and rural areas was found (Table 3).

## 4. Discussion

To the best of our knowledge, this is the *first* study to describe quantitatively axial length differences between parents and their children, as well as its risk factors, in both the urban and rural areas of China. There were several noteworthy findings in the current study. First, the ALD as well as the proportion of children with longer AL than their parents increased as the children's age increased in both the urban and rural areas, similar to our previous reports on refractive error change [5, 6]. Furthermore, the vitreous chamber depth showed the greatest change in the urban children, thus indicating that posterior segment elongation was an important reason for myopic progression in children with moderate refractive error (mean −3.49D and −1.28D for urban and rural children aged 16–17 years, respectively). Second, using an estimation model similar to that used for refractive error change [5, 6], the rural children's AL would be close to the average AL of their parents 2 years earlier than that in the urban children (9 and 11 years old, respectively). However, the estimated generational axial length shift in the urban area would be 1.53 mm, thus approximately 1 mm more than the predicted in the rural area (0.57 mm). Third, older children and males had larger ALD in both urban and rural areas. This was comparable to Saw et al.'s study [8]. They found that AL was associated with older age and being male [8]. Lastly, in the present study, children with more educated parents and more outdoor activity had less ALD in the urban area. It was widely reported that children with less outdoor activity may have less myopic refraction [19–23] and thus may have a shorter axial length and less axial length increase as compared to their parents observed in this study. The higher education level may be correlated with more myopic refraction and longer axial length in the parents [24, 25] and thus with less ALD in this study. However, perhaps due to the less parental education in the rural area (most were junior middle school or less), this association was not observed.

People in the nearby regions of the country (northern China) share extremely close genetic backgrounds, including myopigenic ones. Hence, these children would be predicted to develop similar axial lengths, if there were no other factors involved. Before about the age of 11 years, the rural ALD fitting curve was above the urban one (Figure 1). This may be due to the shorter axial length in their respective parents (mean: urban 24.04 mm vs. rural 22.93 mm). The urban children are putatively exposed to more intensive near work and less outdoor activity as compared to the rural children from an early age [26]. In the current study, children who spent less time in outdoor activity had a larger ALD in the urban area, thus suggesting that outdoor activity time could have a delayed effect on axial length growth. However, no such effect was found in the rural area. We speculate that this may be due to a reduced near work load, as well as more widely spread housing, in the rural area [15]. Although not significant, urban children read more books per week (>2 books/week, 28.9% vs. 6.7%) and lived in more crowded spaces than the rural children (e.g., 88.4% of the urban children have high buildings in front of their house as compared to only 11.1% in the rural children) (Table 3).

The current study provides further evidence for the predominant cohort effect on myopia development. Epidemiological studies have found a remarkably increasing trend in the prevalence of myopia in the same area decades later in both children [27–29] and adults [29–31]. In our previous studies, there was an increasing trend of myopic refraction from parents to their children. The increasing trend was confirmed in the current study by the correlated axial length parameter. Parental axial length in the urban and rural areas did not change significantly as their age increased (mean age: urban 40.0 ± 3.5 years, rural 36.5 ± 4.4 years; every 5 years, *β* = 0.10, *p*=0.16; *β* = −0.03, *p*=0.32). Hence, this trend of refraction and axial length between generations may not be attributed to a physiological decrease of ocular biometric parameters in adults younger than 45 years but predominantly due to increased exposure to myopigenic environmental factors between the two generations [5, 6].

There were some potential limitations of this study. First, the representativeness of the urban area may be limited, since the BMPS was a hospital-based study. Second, there was a likely undersampling in some age groups. Third, only children aged 6 to 17 years were enrolled for this study. It would have been optimal to include young adults greater than 18 years of age as well, which will help to predict even better the generational axial length shift. Hence, further studies with a larger sample and wider age range are warranted.

In summary, this study found different axial length developmental patterns between rural and urban children in China. The estimated generational axial length shift was approximately 1 mm more in the urban children compared to the urban cohort. Environment factors, such as parental education level and outdoor activity, may influence the axial length difference between the two generations.

## Figures and Tables

**Table 1:** Characteristics of children and their parents in the urban and rural areas.

	Children		Parents	
	Urban (*n* = 237)	Rural (*n* = 380)	Urban (*n* = 234)	Rural (*n* = 234)
Number of families	234	234	234	234
Gender (male: female)	121 : 116	210 : 170	—	—
Age (year)	10.0 ± 3.0	10.7 ± 2.5	F: 41.2 ± 4.0	F: 36.7 ± 4.4
			M: 38.8 ± 3.6	M: 36.3 ± 4.5
Spherical equivalent (diopter)	−1.48 ± 2.42	−0.06 ± 1.26	−2.34 ± 1.82	−0.57 ± 0.78
ACD (mm)	3.59 ± 0.27	3.69 ± 0.24	3.25 ± 0.24	3.04 ± 0.27
LT (mm)	3.58 ± 0.21	3.51 ± 0.18	4.25 ± 0.21	4.33 ± 0.30
VCD (mm)	16.69 ± 1.24	15.88 ± 0.78	16.56 ± 0.90	15.56 ± 0.55
AL (mm)	23.86 ± 1.28	23.08 ± 0.81	24.04 ± 0.94	22.93 ± 0.54

F: fathers; M: mothers; ACD: anterior chamber depth; LT: length thickness; VCD: vitreous chamber depth; AL: axial length. Children's ocular parameters were defined as the average of the right eyes; parental ocular parameters were defined as the average of the right eyes of the father and mother.

**Table 2:** Ocular parameter differences (mean ± standard, mm) by children's age in the urban and rural areas.

Children's age (year)	Urban					Rural				
	*N*	ACDD	LTD	VCDD	ALD	*N*	ACDD	LTD	VCDD	ALD
6∼	68	0.21 ± 0.28	−0.56 ± 0.22	−0.62 ± 0.98	−0.96 ± 1.02	39	0.51 ± 0.29^†^	−0.70 ± 0.31^*∗*^	−0.13 ± 0.72^†^	−0.32 ± 0.74^†^
8∼	62	0.26 ± 0.28	−0.67 ± 0.25	−0.37 ± 0.98	−0.78 ± 1.06	88	0.64 ± 0.32^†^	−0.80 ± 0.29^†^	0.10 ± 0.61^†^	−0.05 ± 0.65^†^
10∼	23	0.41 ± 0.28	−0.72 ± 0.25	0.46 ± 1.10	0.15 ± 1.12	117	0.64 ± 0.29^†^	−0.82 ± 0.28	0.35 ± 0.63	0.17 ± 0.63
12∼	53	0.44 ± 0.26	−0.73 ± 0.18	0.72 ± 0.94	0.48 ± 0.91	86	0.67 ± 0.30^†^	−0.88 ± 0.41^†^	0.59 ± 0.88	0.38 ± 0.90
14∼	16	0.59 ± 0.32	−0.77 ± 0.26	1.65 ± 1.04	1.44 ± 1.16	35	0.78 ± 0.25^*∗*^	−0.85 ± 0.31	0.51 ± 0.78^†^	0.44 ± 0.74^†^
16–17	15	0.54 ± 0.31	−0.76 ± 0.19	1.39 ± 0.96	1.17 ± 0.97	15	0.74 ± 0.31	−0.84 ± 0.24	0.67 ± 0.71^*∗*^	0.57 ± 0.73
*p* for trend		**<0.001**	**<0.001**	**<0.001**	**<0.001**		**<0.001**	**0.024**	**<0.001**	**<0.001**

ACDD: central anterior chamber depth difference; LTD: length thickness difference; VCDD: vitreous chamber depth difference; ALD: axial length difference. ^*∗*^*p* < 0.05 compared to corresponding urban data; ^†^*p* < 0.001 compared to corresponding urban data. Bold values are statistically significant.

**Table 3:** Age- and gender-adjusted association between axial length difference (ALD, mm) and risk factors.

	Urban				Rural			
	*N*	ALD (mean ± SD)	Adjusted *β* coefficient	*p*	N	ALD (mean ± SD)	Adjusted *β* coefficient	*p*
Age, year^a^								
6∼	68	−0.96 ± 1.02	Ref	Ref	39	−0.32 ± 0.74	Ref	Ref
8∼	62	−0.78 ± 1.06	0.23	0.20	88	−0.05 ± 0.65	0.26	**0.04**
10∼	23	0.15 ± 1.12	1.16	**<0.001**	117	0.17 ± 0.63	0.49	**<0.001**
12∼	53	0.48 ± 0.91	1.47	**<0.001**	86	0.38 ± 0.90	0.78	**<0.001**
14∼	16	1.44 ± 1.16	2.43	**<0.001**	35	0.44 ± 0.74	0.92	**<0.001**
16–17	15	1.17 ± 0.97	2.10	**<0.001**	15	0.57 ± 0.73	1.01	**<0.001**
Trend test			0.26	**<0.001**			0.11	**<0.001**

Gender^b^								
Male	121	0.08 ± 1.29	Ref	Ref	210	0.34 ± 0.71	Ref	Ref
Female	116	−0.46 ± 1.26	−0.55	**<0.001**	170	−0.08 ± 0.76	−0.52	**<0.001**

Father's education^c^								
1st tertile (lowest)	59	−0.14 ± 1.32	Ref	Ref	112	0.15 ± 0.73	Ref	Ref
2nd tertile	134	−0.16 ± 1.37	−0.49	**0.002**	224	0.17 ± 0.72	−0.07	0.39
3rd tertile	44	−0.32 ± 1.05	−0.58	**0.003**	33	0.07 ± 1.13	−0.23	0.09
Trend test			−0.30	**0.002**			−0.10	0.11

Mother's education^c^								
1st tertile (lowest)	57	−0.06 ± 1.25	Ref	Ref	229	0.13 ± 0.72	Ref	Ref
2nd tertile	126	−0.19 ± 1.38	−0.44	**0.005**	129	0.14 ± 0.77	−0.01	0.92
3rd tertile	44	−0.32 ± 1.05	−0.55	**0.005**	14	0.43 ± 1.18	0.14	0.49
Trend test			−0.29	**0.004**			0.02	0.74

Near work activity (h/d)								
1st tertile (lowest)	76	−0.29 ± 1.26	Ref	Ref	122	−0.03 ± 0.63	Ref	Ref
2nd tertile	73	−0.01 ± 1.36	0.00	0.99	132	0.22 ± 0.82	0.10	0.25
3rd tertile	72	−0.11 ± 1.28	−0.31	0.06	125	0.27 ± 0.79	0.13	0.15
Trend test			−0.16	0.06			0.06	0.15

Outdoor activity (h/d)								
1st tertile (lowest)	73	0.21 ± 1.26	Ref	Ref	131	0.12 ± 0.73	Ref	Ref
2nd tertile	76	−0.22 ± 1.24	−0.21	0.20	122	0.13 ± 0.75	−0.06	0.52
3rd tertile	72	−0.49 ± 1.31	−0.45	**0.006**	126	0.23 ± 0.80	0.02	0.86
Trend test			−0.23	**0.006**			0.01	0.86

Books read per week (number)								
≤2	138	−0.14 ± 1.25	Ref	Ref	196	0.15 ± 0.75	Ref	Ref
>2	56	−0.23 ± 1.36	0.10	0.53	14	0.62 ± 0.70	0.37	0.06

Continuous reading (minutes)^d^								
0–15	44	−0.09 ± 1.37	Ref	Ref	62	−0.01 ± 0.74	Ref	Ref
16–30	49	−0.20 ± 1.44	−0.07	0.72	157	0.15 ± 0.71	0.12	0.24
31–45	56	−0.36 ± 1.15	−0.11	0.57	121	0.16 ± 0.80	0.09	0.38
>45	76	−0.02 ± 1.26	−0.19	0.31	39	0.44 ± 0.82	0.23	0.10
Trend test			−0.06	0.30			0.05	0.20

High buildings in front of the house								
No	23	−0.31 ± 1.09	Ref	Ref	327	0.14 ± 0.75	Ref	Ref
Yes	175	−0.17 ± 1.29	−0.04	0.85	41	0.24 ± 0.85	0.05	0.65

Horizon in front of the house								
No	123	−0.25 ± 1.19	Ref	Ref	249	0.13 ± 0.77	Ref	Ref
Yes	45	−0.55 ± 1.18	0.01	0.96	105	0.23 ± 0.75	0.02	0.80

SD: standard deviation; Ref: reference group; h/d: hour/day; values in bold denote statistical significance. ^a^only gender was adjusted; ^b^only age was adjusted; ^c^for urban area, education level was senior school and less, college, master and above, respectively; for rural area, education level was primary school and less, junior school, senior school and above, respectively; ^d^defined as time spent on continuous reading before taking a break of 5 minutes or longer.

## Data Availability

The data used to support the findings of this study are available from the corresponding author upon request.
